# Chinese medical named entity recognition based on multimodal information fusion and hybrid attention mechanism

**DOI:** 10.1371/journal.pone.0325660

**Published:** 2025-06-11

**Authors:** Zhen Luo, Jingping Che, Fan Ji

**Affiliations:** School of Artificial Intelligence, Zhoukou Normal University, Zhoukou, Henan, China; Air Force Engineering University, CHINA

## Abstract

Chinese Medical Named Entity Recognition (CMNER) seeks to identify and extract medical entities from unstructured medical texts. Existing methods often depend on single-modality representations and fail to fully exploit the complementary nature of different features. This paper presents a multimodal information fusion-based approach for medical named entity recognition, integrating a hybrid attention mechanism. A Dual-Stream Network architecture is employed to extract multimodal features at both the character and word levels, followed by deep fusion to enhance the model’s ability to recognize medical entities. The Cross-Stream Attention mechanism is introduced to facilitate information exchange between different modalities and capture cross-modal global dependencies. Multi-Head Attention is employed to further enhance feature representation and improve the model’s ability to delineate medical entity boundaries. The Conditional Random Field (CRF) layer is used for decoding, ensuring global consistency in entity predictions and thereby enhancing recognition accuracy and robustness. The proposed method achieves F1 scores of 65.26%, 80.31%, and 86.73% on the CMeEE-V2, IMCS-V2-NER, and CHIP-STS datasets, respectively, outperforming other models and demonstrating significant improvements in medical entity recognition accuracy and multiple evaluation metrics.

## 1. Introduction

Chinese Medical Named Entity Recognition (CMNER) is a crucial task in Natural Language Processing (NLP) within the medical domain. Its objective is to automatically identify and extract medical entities, including diseases, symptoms, drugs, surgeries, and organisms, from unstructured medical texts. [1] This task has significant applications in areas such as medical information extraction, clinical decision support, and medical knowledge graph construction. However, due to the specialized nature and complexity of medical texts, as well as the linguistic characteristics of Chinese, CMNER faces numerous challenges, and existing methods still require improvements in accuracy and robustness.

In medical text processing, CMNER primarily involves extracting medical entities from sources such as Electronic Medical Records (EMRs), medical literature, and clinical notes, thereby providing reliable data support for downstream medical information processing. [2] Compared with general Named Entity Recognition (NER), medical NER presents the following challenges:

1) The absence of explicit word boundaries in Chinese text leads to ambiguity in word-level information of varying granularities. Medical entities are often composed of multiple subwords, which are easily affected by word segmentation errors, resulting in inaccurate entity boundary recognition. For example, “急性心肌梗死” may be incorrectly segmented into “急性/心肌/梗死” or “急性心肌/梗死”, which affects the correct recognition of entities.
2) The medical domain encompasses a wide range of entity types, and identical words in different contexts may correspond to distinct entity categories. For example, “发烧” may be a symptom, while “病毒性发烧” may refer to a disease. Some entity names may have different meanings in different medical scenarios, which increases the difficulty of recognition.
3) Most existing CMNER methods rely on single-modality features (e.g., character-level or word-level features) for entity recognition, neglecting the complementary nature of different levels of information, such as characters, words, and radicals, in medical texts. Although the traditional BiLSTM-CRF-based method can model the contextual information of the text, it fails to fully utilize the semantic relationship between multimodal features, limiting the expressive power of the model.
4) Medical texts are often lengthy, exhibiting long-range dependencies among medical entities. In clinical reports, disease descriptions and treatment plans may be separated by multiple sentences, and traditional RNN structures are difficult to effectively capture long-distance dependency information. How to model long-distance dependencies in CMNER tasks is a key issue in improving entity recognition performance.

At present, traditional medical named entity recognition methods mainly rely on medical dictionaries and rule matching, regular expression matching, pattern rule matching, etc. These methods have high interpretability, but in practical applications, they have problems such as poor generalization ability and inability to recognize new entities. Machine learning methods, hidden Markov models (HMM [3]) and conditional random fields (CRF [4]), perform entity recognition by manually constructing features. These methods are highly dependent on the construction of artificial features, cannot fully utilize the potential information of large-scale medical texts, and are difficult to adapt to complex entity recognition tasks. In recent years, deep learning methods have been widely used in CMNER tasks, among which the BiLSTM-CRF structure has become one of the mainstream methods. This method combines the bidirectional long short-term memory network (BiLSTM [5]) to extract contextual features and uses the conditional random field (CRF) to decode entities, thereby improving the global consistency of recognition. This method only relies on character-level information and lacks effective fusion of multimodal features. It is difficult to capture long-distance dependencies in long texts. Limited by the sequence modeling method of the RNN structure, it is difficult to parallelize calculations, which affects the training efficiency of the model.

To address these challenges, this paper proposes a CMNER method incorporating multimodal information fusion and a hybrid attention mechanism to fully exploit the multi-level features of medical texts, thereby enhancing the accuracy and robustness of entity recognition. The method of this paper mainly includes the following core modules:

1) Multimodal feature fusion using a Dual-Stream Network: This structure extracts multimodal features from two levels—character and word—and integrates them deeply to enhance the semantic representation of medical texts.
2) Cross-Stream Attention Mechanism: Features extracted at the character and word levels interact through cross-stream attention, facilitating global dependency learning and mitigating the limitations of single-modality information.
3) Multi-Head Attention Mechanism: This mechanism enhances the model’s capacity to recognize medical entity boundaries and improves long-text representation.
4) GGNN for Entity Dependency Modeling: The Gated Graph Neural Network (GGNN) models entity dependencies in medical texts, capturing structured connections among entities and enhancing both entity boundary recognition and overall performance.

## 2. Related work

Research on Chinese Medical Named Entity Recognition (CMNER) primarily focuses on integrating character-level and word-level features. Existing studies still exhibit limitations in multimodal information fusion, cross-level feature interaction, and the mitigation of word segmentation errors. To address these challenges, researchers have explored rule-based methods, traditional machine learning approaches, deep learning techniques, and graph neural networks for enhancement.

### 2.1. Rule-based and machine learning methods

Early methods for medical named entity recognition primarily relied on rule-based and machine learning approaches. Perera et al. [6] proposed a machine learning approach for medical entity recognition based on a hidden Markov model (HMM), utilizing hand-designed character-level and word-level features. Li et al. [7] combined conditional random fields (CRF) to investigate the role of various feature types in Chinese medical named entity recognition. Venkataraman et al. [8] developed a medical terminology dictionary and integrated it with regular expressions to recognize medical entities. Ke et al. [9] employed a pattern-matching-based strategy to enhance the accuracy of medical entity recognition by constructing a medical concept hierarchy dictionary. However, this method performed poorly when encountering new and out-of-vocabulary (OOV) words. Li et al. [10] further refined the dictionary-based matching strategy by incorporating coreference resolution techniques to enhance the recognition of synonymous entities. An et al. [11] attempted to integrate statistical learning methods with dictionary matching to enhance the recognition of medical entities through joint probability modeling. However, this method still depends on manual features and lacks automatic learning capabilities. Chen et al. [12] combined semi-supervised learning, utilizing a small amount of labeled data and a large amount of unlabeled data to train the model, thereby improving the generalization ability of medical entity recognition.

### 2.2. Methods based on deep learning

With the advancement of deep learning, an increasing number of researchers are using neural networks for medical entity recognition to enhance the model’s feature representation capability and generalization performance. Zhang et al. [13] proposed a CNN-LSTM-CRF model that employs a convolutional neural network (CNN) to extract local features, thereby enhancing the accuracy of medical entity recognition. Huang et al. [14] proposed a character-word LSTM model to improve the model’s recognition of medical entities. Zhang et al. [15] effectively mitigated the impact of word segmentation errors on named entity recognition by integrating the lattice structure with position encoding. Beebe et al. [16] employed a Tree-LSTM structure to integrate character, word, and lattice information for medical entity recognition, enabling effective modeling of long-distance dependencies. Shi et al. [17] designed a method that integrates a multi-layer convolutional neural network (CNN) with an attention mechanism to capture text features at various levels. Liu et al. [18] enhanced the performance of medical entity recognition by integrating text context representation with Chinese character shape information and utilizing character morphological knowledge. Yang et al. [19] employed the Transformer structure in combination with the multi-head attention mechanism to enhance the accuracy of medical entity recognition. Zheng et al. [20] proposed a cross-modal feature fusion method that integrates character, word, pinyin, and radical information to further enhance the effectiveness of medical entity recognition. Arslan et al. [21] integrated the pre-trained language model BERT with the BiLSTM-CRF structure to enhance the context modeling ability of medical entity recognition. Gong et al. [22] employed a graph neural network (GNN) to enhance the recognition of complex entities by modeling the dependency relationships between entities, further optimizing the performance of medical named entity recognition. Wang et al. [23] proposed a method combining multi-granularity semantic dictionaries and multimodal trees to improve recognition accuracy by fusing semantic information of different granularities. Sun et al. [24] further enhanced the model’s expressiveness in complex medical texts by fusing global features with multiple local features. In the field of adversarial learning, the EVADE study proposed by Tian et al. [25] on adversarial false data injection attack methods for smart grid state estimation reveals potential threats in infrastructure security. The LESSON technology developed by Tian et al. [26] improves the location and detection capabilities of such attacks through multi-label deep learning methods. These studies not only expand the technical boundaries of their respective fields, but also provide valuable methodological references for medical text processing and intelligent system security protection, inspiring us to pay more attention to the diversity of feature representation and the construction of model anti-interference capabilities when designing medical named entity recognition models.

### 2.3. Application of graph neural networks in CMNER tasks

In recent years, the application of graph neural networks (GNNs) in named entity recognition (NER) tasks has garnered significant attention, particularly in the medical field. GNNs can effectively model the dependency relationships between entities, thereby enhancing the accuracy of entity recognition. Gu et al. [27] extracted syntactic dependency information using graph convolutional networks (GCNs), significantly enhancing the global representational capacity of the model. Zhang et al. [28] proposed a multi-graph collaborative graph attention network (CGN) to enhance the interaction of information between multi-graph structures, enabling the model to more effectively aggregate feature information from diverse perspectives. Building on this, Niu et al. [29] further integrated lexical, character, and word-level information to enhance the performance of medical entity recognition. Han et al. [30] proposed a syntactically enhanced BiLSTM-CRF model based on GCN to improve the contextual representation of medical entities. Ma et al. [31] incorporated medical dictionaries to encode words and positional information during the graph structure construction process, thereby improving the accuracy of medical entity recognition. Boutsikaris et al. [32] employed a dual-channel GCN structure to simultaneously model word-level and character-level dependencies, integrating a self-attention mechanism to enhance global feature representation capabilities. Du et al. [33] proposed a GNN-based entity boundary enhancement method to mitigate the issue of misjudging entity boundaries by the traditional BiLSTM-CRF structure. Sengupta et al. [34] integrated the BERT pre-trained model with the GCN structure to achieve strong performance in medical entity recognition in low-resource settings. Although the current CMNER method has made some progress in medical entity recognition, it still has several limitations. Many methods fail to effectively model the semantic relationships between adjacent characters, making the model prone to errors when processing entity boundaries. Current models seldom combine lexical features (such as character-level and word-level features) with syntactic features (such as dependencies between entities), leading to inaccuracies in entity boundary detection and category classification. To address these issues, this paper proposes a medical named entity recognition method based on multimodal information fusion and a hybrid attention mechanism to optimize the performance of the CMNER task.

## 3. Method

**Fig 1:**
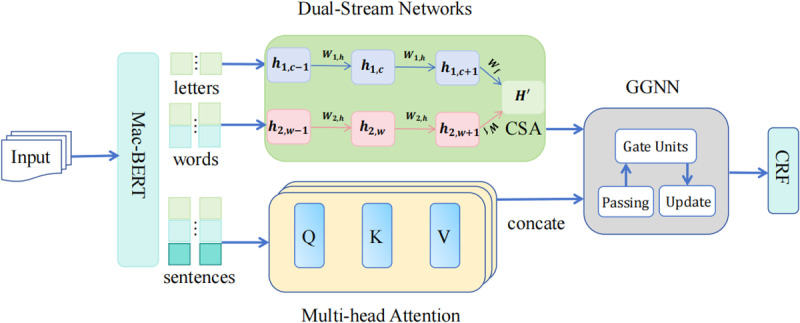
The proposed model architecture.

In response to the challenges of word segmentation errors, ambiguous entity boundaries, and the absence of multimodal information fusion in the Chinese Medical Named Entity Recognition (CMNER) task, this paper presents a medical named entity recognition model based on multimodal information fusion and a hybrid attention mechanism. This method leverages character-level and word-level features, performs deep fusion via Dual-Stream Networks, and integrates cross-stream attention mechanisms (Cross-Stream Attention) and multi-head attention mechanisms (Multi-Head Attention) to facilitate feature interaction and enhance feature representation. The model employs a Gated Graph Neural Network (GGNN) to model medical entity dependencies and decodes the sequence through a conditional random field (CRF) to ensure the global consistency of the predicted sequence. The model architecture proposed herein is shown in Fig 1:

As illustrated in Fig 1, the proposed model consists of the following key modules:

1) MacBERT-based character-level and word-level feature extraction: The MacBERT pre-trained model is utilized to extract character-level representations, while word-level information is acquired through dictionary matching, thereby enhancing the model’s text representation capabilities.
2) Dual-Stream Networks for multimodal feature fusion: The character-level and word-level features are encoded independently, with the cross-stream attention mechanism (CSA) facilitating information interaction between the two modalities.
3) Multi-Head Attention for entity boundary modeling: This module enhances the model’s ability to recognize medical entity boundaries and improves its global feature expression.
4) GGNN for entity dependency modeling: The Gated Graph Neural Network (GGNN) is employed to model the grammatical dependencies between entities, optimizing the entity recognition process.
5) CRF for global sequence decoding: This module optimizes the coherence of the prediction results, ensuring the accuracy and stability of entity recognition.

### 3.1. Character-level and word-level feature extraction based on MacBERT

#### 3.1.1. Semantic representation of medical text.

In the medical domain, texts often include numerous specialized terms and specific expressions. Choosing an appropriate pre-trained model as a text encoder is crucial. This study employs MacBERT as a text encoder to enhance the learning of semantic information in the medical domain. MacBERT (Masked Language Model for Correction of BERT) is a pre-trained model built on the BERT architecture. It addresses the potential bias in BERT’s Masked Language Model task by employing a masking strategy based on synonym replacement (MLM as correction, Mac), thereby improving the model’s semantic understanding of text.

MacBERT utilizes the Transformer architecture, centered around the self-attention mechanism and the feed-forward neural network (FFN). The self-attention mechanism allows each character to attend to all other characters in the text simultaneously during representation calculation, thereby fully capturing contextual information. FFN further applies nonlinear transformations to the output of the self-attention mechanism, enhancing the model’s representational capacity. MacBERT employs a bidirectional encoding strategy, allowing the model to incorporate information from both the left and right directions during text encoding, thereby achieving a more accurate understanding of medical terms.

In the medical named entity recognition task, MacBERT, as a fundamental encoder, effectively learns the contextual information of medical entities, thereby improving the model’s ability to recognize complex medical terms. Given an input text sequence $X=\{x_{1},x_{2},\ldots ,x_{n}\}$, the representation after encoding through MacBERT is:


$$H_{c}=MacBERT(X)=\{h_{1}^{c},h_{2}^{c},\ldots ,h_{n}^{c}\}$$ (1)

Where X represents the input medical text sequence; $H_{c}\in {\mathbb{R}}^{n\times d}$ represents the word-level representation output by MacBERT; $h_{i}^{c}\;$ is the hidden state representation of the i-th word; d is the hidden layer dimension; after encoding by MacBERT, the obtained word-level features provide rich contextual information for subsequent entity recognition tasks, allowing the model to more accurately judge entity boundaries.

#### 3.1.2. Word-level information extraction and modeling.

Many entities in medical texts consist of multiple characters, such as “chronic obstructive pulmonary disease” and “aspirin”, making it insufficient to rely solely on word-level features for accurate entity identification. This paper further incorporates word-level features to address the limitations of character-level representation. Word-level feature acquisition is based on the SoftLexicon mechanism. As entities in medical texts are often lengthy, traditional word segmentation tools may missegment them, while the SoftLexicon mechanism effectively alleviates this issue. Medical proper nouns frequently exhibit specific lexical collocation patterns. By integrating word-level features, the model can more effectively learn these patterns and enhance the accuracy of entity recognition. The SoftLexicon mechanism searches for potential words in the input text using dictionary matching with a medical terminology dictionary and constructs word-level embeddings. Given the input text $X=\{x_{1},x_{2},\ldots ,x_{n}\}$, its word-level representation is:


$$H_{w}=Lexicon(X)=\{h_{1}^{w},h_{2}^{w},\ldots ,h_{m}^{w}\}$$ (2)

Where $H_{w}\in {\mathbb{R}}^{m\times d}$ represents the matched word-level representation; $h_{j}^{w}$ is the representation of the j-th matched word; and m is the number of matched words.

#### 3.1.3. Word feature fusion.

To fully utilize both character-level and word-level information, this paper combines the features of these two modalities to create a comprehensive input representation. The final fused feature representation is given by:


$$H=[H_{c};H_{w}]$$ (3)

Where $H\in {\mathbb{R}}^{n\times 2d}$ represents the fused feature representation. By concatenating character-level and word-level embeddings, the model can incorporate information from both the character and lexical levels, thereby enhancing the performance of medical entity recognition. By jointly modeling character-level and word-level features, the proposed method can more comprehensively capture the semantic information of medical texts and provide a more robust feature representation for subsequent named entity recognition tasks.

### 3.2. Dual-stream networks for multimodal feature fusion

In the task of Medical Named Entity Recognition (CMNER), character-level and word-level information each have distinct expressive capabilities. Character-level information captures semantic features in a fine-grained manner, while word-level information conveys the overall meaning of medical terms. To fully utilize the information from these two modalities, this paper employs Dual-Stream Networks for multimodal feature fusion and enhances the interaction between different modalities through the Cross-Stream Attention mechanism, thereby improving the accuracy and stability of entity recognition.

#### 3.2.1. Dual-stream network structure.

Dual-Stream Networks utilizes two independent data streams to process character-level and word-level information, and encodes character-level features $H_{c}$ and word-level features $H_{w}$ independently:


$$H_{c}^{\prime }=f_{c}\left(H_{c}\right),H_{w}^{\prime }=f_{w}(H_{w})$$ (4)

Where $f_{c}$ and $f_{w}$ represent the feature conversion functions at the character level and word level, respectively; $H_{c}^{\prime }\in {\mathbb{R}}^{n\times d}$ is the converted character-level representation; $H_{w}^{\prime }\in {\mathbb{R}}^{m\times d}$ is the converted word-level representation. In this structure, character-level features capture fine-grained information from medical texts, while word-level features provide additional semantic enhancement via dictionary matching. After the two data streams are processed independently, feature interaction is necessary to fully leverage the information from both modalities.

#### 3.2.2. Cross-stream attention mechanism.

To enhance the interaction between character-level and word-level features, this study adopts the Cross-Stream Attention mechanism, which allows the character stream to learn information from the word stream and computes the query, key, and value for both streams, respectively:


$$\begin{array}{c} Q_{c}=W_{q}H_{c}^{\prime },K_{w}=W_{k}H_{w}^{\prime },V_{w}=W_{v}H_{w}^{\prime }\;\\ Q_{w}=W_{q}H_{w}^{\prime },K_{c}=W_{k}H_{c}^{\prime },V_{c}=W_{v}H_{c}^{\prime } \end{array}$$ (5)

Where $W_{q},W_{k},W_{v}$ are a learnable parameter; $Q_{c},K_{c},V_{c}$ represents the query, key, and value of the character stream; and y represents the query, key, and value of the word stream. The attention weights from the character stream to the word stream and from the word stream to the character stream are then computed as follows:


$$\begin{array}{c} A_{c}=softmax\left(\frac{Q_{c}K_{w}^{T}}{\sqrt{d}}\right)V_{w}\;\\ A_{w}=softmax\left(\frac{Q_{c}K_{c}^{T}}{\sqrt{d}}\right)V_{c} \end{array}$$ (6)

Where $A_{c}\in {\mathbb{R}}^{n\times d}$ is the feature obtained after the character-level representation is integrated with the word-level information; $A_{w}\in {\mathbb{R}}^{m\times d}$ is the feature after the word-level representation is integrated with the character-level information; d is the hidden layer dimension; softmax normalization ensures that the weight sum is 1 to enhance the feature selection ability. The fused feature representation is given by:


$$H^{\prime }=[A_{c};A_{w}]$$ (7)

Where $H^{\prime }\in {\mathbb{R}}^{(n+m)\times d}$ is the fused representation vector. Through the cross-stream attention mechanism, the character and word streams can complement each other, improving the model’s accuracy in entity boundary recognition.

### 3.3. Multi-head attention mechanism and GGNN dependency modeling

After the feature fusion of Dual-Stream Networks is completed, the resulting word fusion features remain local and lack the ability to model global entity dependencies. To further enhance entity boundary recognition and capture global dependencies between medical terms, this paper employs the Multi-Head Attention mechanism for feature enhancement and utilizes the Gated Graph Neural Network (GGNN) to model the syntactic dependencies between entities, thereby improving the recognition of medical entities.

#### 3.3.1. Multi-head attention mechanism.

The Multi-Head Attention mechanism (MHA) is an attention mechanism that improves feature expression capabilities. It processes information in parallel across different subspaces through multiple attention heads and combines them to improve the model’s learning ability. Given the fused feature $H^{\prime }$ processed by the Dual-Stream Networks, the multi-head attention defines the query, key, and value matrices:


$$Q=W_{q}H^{\prime },K=W_{k}H^{\prime },V=W_{v}H^{\prime }$$ (8)

Where $W_{q},W_{k},W_{v}$ are the learnable parameter matrix; Q,K,V represents the transformed features of query, key and value respectively. Scaled Dot-Product Attention is computed for each attention head:


$${head}_{i}=Attention(QW_{q}^{i},KW_{k}^{i},VW_{v}^{i})$$ (9)

$$Attention(Q,K,V)=softmax\left(\frac{QK^{T}}{\sqrt{d}}\right)V$$ (10)

Where d is the hidden layer dimension, $\sqrt{d}$ serves as a scaling factor to ensure gradient stability. Softmax normalization ensures the probability distribution of attention weights, concatenates the outputs of all attention heads, and obtains the final multi-head attention output after linear transformation:


$$H^{\prime \prime }=Concat({head}_{1},\ldots ,{head}_{h})W_{o}$$ (11)

Where h represents the number of attention heads; $W_{o}$ is the learnable projection matrix; and $H^{\prime \prime }\in {\mathbb{R}}^{(n+m)\times d}$ represents the fused representation vector.

#### 3.3.2. GGNN for entity dependency modeling.

Although the multi-head attention mechanism has enhanced the model’s feature expression capabilities, it remains a sequence-based modeling method, making it challenging to directly use the grammatical dependencies between entities. To further model the structured dependency information between medical terms, this study employs the Gated Graph Neural Network (GGNN) for entity relationship modeling. The core idea of GGNN is to utilize the Gated Recurrent Unit (GRU) for graph neural network propagation to effectively model long-distance dependencies. Given the hidden state $H_{t}$ of the t-th layer, GGNN calculates the normalization of the adjacency matrix:


$$\tilde{A}=D^{-\frac{1}{2}}AD^{-\frac{1}{2}}$$ (12)

Where D is the degree matrix of the adjacency matrix A, followed by the calculation of graph neural network propagation:


$${\tilde{H}}_{t}=\tilde{A}H_{t}W_{g}$$ (13)

Where $W_{g}$ is the learnable transformation matrix; ${\tilde{H}}_{t}$ is the feature after propagation through the adjacency matrix, and GRU is used for gated update:


$$\begin{array}{c} Z_{t}=\sigma \left(W_{z}{\tilde{H}}_{t}+U_{z}H_{t-1}\right)\\ \begin{array}{c} R_{t}=\sigma (W_{r}{\tilde{H}}_{t}+U_{r}H_{t-1}\backslash \backslash \begin{array}{c} {\hat{H}}_{t}=\tanh\left(W_{h}{\tilde{H}}_{t}+R_{t}⊙U_{h}H_{t-1}\right)\\ H_{t}=\left(1-Z_{t}\right)⊙H_{t-1}+Z_{t}⊙{\hat{H}}_{t} \end{array} \end{array} \end{array}$$ (14)

Where $Z_{t}$ is the update gate, which determines the degree of retention of historical information in the current state; $R_{t}$ is the reset gate, which controls the impact of the current input on historical information; $W_{z},W_{r},W_{h},U_{z},U_{r},U_{h}$ is a learnable parameter; ⊙ represents element-by-element multiplication; σ is the Sigmoid function, and tanh is the Tanh activation function. After T rounds of GGNN propagation, the final output of entity dependency modeling is obtained:


$$H_{GGNN}=H_{T}$$ (15)

Where $H_{T}\in {\mathbb{R}}^{(n+m)\times d}$ represents the final representation that incorporates the dependencies.

### 3.4. CRF for sequence decoding

After multimodal feature fusion by the Dual-Stream Networks and entity dependency modeling using the Multi-Head Attention (MHA) mechanism and the Gated Graph Neural Network (GGNN), the model derives the final feature representation $H_{final}$ of the medical text. In the Named Entity Recognition (NER) task, independently predicting the label of each word or phrase in isolation may result in an inconsistent prediction sequence. To ensure global consistency in entity label sequences, this study employs a Conditional Random Field (CRF) for sequence annotation decoding, enhancing the coherence of predictions and improving both the accuracy and stability of entity recognition.

#### 3.4.1. CRF structure and mathematical modeling.

The Conditional Random Field (CRF) is a probabilistic graphical model designed to capture label dependencies across an entire sequence. Given the final representation $H_{final}$ of the input sequence, the CRF aims to predict the optimal label sequence $Y=\{y_{1},y_{2},\ldots ,y_{n}\}$ through global optimization. Define the state transfer matrix $T\in {\mathbb{R}}^{\left|L|\times |L\right|}$, where |L| is the total number of entity label categories; $T_{i,j}$ represents the score of transferring from label i to label j. For a given input X and label sequence Y, the CRF score function is defined as follows:


$$S(X,Y)=\sum_{i=1}^{n}P_{i}(y_{i})+\sum_{i=1}^{n-1}T_{y_{i},y_{i+1}}$$ (16)

Here, $P_{i}$ denotes the score assigned by the model to the i-th character or word assigned to label $y_{i}$ from the model output; $T_{y_{i},y_{i+1}}$ represents the transition probability from label $y_{i}$ to label $y_{i+1}$. The goal of CRF is to find the label sequence that maximizes the global score:


$$Y^{*}=arg\max_{Y}S(X,Y)$$ (17)

Where $Y^{*}$ is the optimal label sequence.

#### 3.4.2. Training process and loss function.

To optimize the CRF structure, we employ the Maximum Log-Likelihood (MLL) training method to ensure that the correct label sequence Y has the highest probability among all possible label sequences $Y^{\prime }$. The normalized probability distribution of CRF is calculated as follows:


$$P\left(Y|X\right)=\frac{exp(S(X,Y))}{\sum_{Y^{\prime }\in y}exp(S(X,Y^{\prime }))}$$ (18)

Here, y denotes the set of all possible label sequences, and the denominator represents the sum of the scores of all possible label sequences, ensuring normalization. The CRF loss function is based on the Negative Log-Likelihood (NLL):


$${ℒ}_{CRF}=-logP\left(Y|X\right)=-\left(S(X,Y)-\log\sum_{Y^{\prime }\in y}exp(S(X,Y^{\prime }))\right)$$ (19)

Here, S(X,Y) denotes the score of the correct label sequence, while $\log\sum_{Y^{\prime }\in y}exp(S(X,Y^{\prime }))$ represents the sum of the scores of all possible label sequences, ensuring probability normalization in the loss function.

## 4. Results

### 4.1. Datasets and evaluation criteria

To evaluate the effectiveness of the proposed method in Chinese medical named entity recognition (CMNER), we conducted experiments using three public medical entity recognition datasets: CMEEE-V2, IMCS-V2-NER, and CHIP-STS. These datasets encompass various types of medical texts, including electronic medical records, clinical dialogues, and medical semantic relationship annotations, enabling a comprehensive evaluation of the model’s generalization ability across different medical contexts.

The CMEEE-V2 (Chinese Medical Event Extraction Evaluation) dataset is specifically designed for Chinese medical event extraction tasks, including annotations for medical named entity recognition (CMNER) and associated event information. This dataset is primarily sourced from electronic medical records (EMRs), medical literature, and clinical reports. It includes annotations for various medical entity categories, such as diseases, symptoms, tests, procedures, and drugs. This dataset is primarily used to assess key medical entities in textual data. The statistics of the CMEEE-V2 dataset are presented in Table 1.

**Table 1:** Entity distribution statistics of CMeEE-V2 dataset.

Data volume	Training set	Validation set	Test Set
23,000	15,000	5,000	3,000

The IMCS-V2-NER dataset consists of medical conversation data collected from the Intelligent Medical Consultation System, encompassing interactions between doctors and patients. The key characteristic of this dataset is its inclusion of colloquial medical texts. Compared to electronic medical records, its textual format is more flexible, and entity expressions are more diverse, making it suitable for evaluating the model’s generalization ability in conversational medical contexts. The statistics of the IMCS-V2-NER dataset are presented in Table 2.

**Table 2:** Entity distribution statistics of the IMCS-V2-NER dataset.

Data volume	Training set	Validation set	Test Set
4,116	2,472	833	811

The CHIP-STS (China Health Information Processing Semantic Text Similarity) dataset is a Chinese dataset designed for evaluating medical semantic relationship matching and entity recognition. This dataset is primarily used for the structured processing of medical texts, includes annotations for multiple medical entity categories, and provides semantic relationship information between entities. Compared to CMEEE-V2 and IMCS-V2-NER, CHIP-STS places greater emphasis on understanding medical entities at the semantic level and imposes higher demands on the model’s capability to represent medical knowledge. The statistics of the CHIP-STS dataset are presented in Table 3.

**Table 3:** Entity distribution statistics of the CHIP-STS dataset.

Data volume	Training set	Validation set	Test Set
21,000	16,000	4,000	1,000

To comprehensively evaluate the performance of medical entity recognition, this study employs precision (P), recall (R), and F1-score as the primary evaluation metrics.


$$\begin{array}{c} P=\frac{TP}{TP+FP}\\ \begin{array}{c} R=\frac{TP}{TP+FN}\;\\ F1=2\times \frac{P\times R}{P+R} \end{array} \end{array}$$ (20)

Among them, TP represents a correctly recognized entity; FP represents an incorrectly recognized entity; and FN represents an unrecognized entity.

### 4.2. Experimental settings

This study performed extensive experiments on three datasets: CMEEE-V2, IMCS-V2-NER, and CHIP-STS. To ensure the reliability of the experimental results, a 5-fold cross-validation method was employed. Each dataset was partitioned into five subsets, four of which were allocated for model training, while the remaining subset was designated for validation. This process was iterated five times for each dataset, with a different subset selected as the validation set in each iteration, while the remaining subsets were used for training. This approach mitigates the influence of data partitioning on the experimental outcomes and enhances the model’s generalization capability.

This study compares the proposed model, M-DSCMG (MacBERT + Dual-Stream Networks + Cross-Stream Attention + Multi-Head Attention + GGNN + CRF), with multiple existing named entity recognition methods on the medical datasets CMEEE-V2, IMCS-V2-NER, and CHIP-STS to validate its effectiveness in Chinese medical named entity recognition. The experimental results are presented in Tables 4–6.

**Table 4:** CMeEE-V2 experimental results.

Model	Precision (%)	Recall (%)	F1 (%)
BERT+CRF	61.22	59.68	60.06
BERT+BiLSTM+CRF	65.31	62.26	63.80
MacBERT+CRF	64.38	61.87	63.46
MacBERTa + Att + CRF	66.36	63.52	64.78
M+DSCMG(Our)	**66.73**	**64.96**	**65.26**

**Table 5:** IMCS-V2-NER experimental results.

Model	Precision (%)	Recall (%)	F1 (%)
BERT+CRF	71.82	75.12	73.36
BERT+BiLSTM+CRF	73.64	74.26	73.93
MacBERT+CRF	78.28	76.36	76.52
MacBERTa + Att + CRF	79.52	77.10	78.30
M+DSCMG(Our)	**81.68**	**79.32**	**80.31**

**Table 6:** CHIP-STS experimental results.

Model	Precision (%)	Recall (%)	F1 (%)
BERT+CRF	83.37	81.71	82.64
BERT+BiLSTM+CRF	84.52	82.06	83.19
MacBERT+CRF	84.76	82.62	83.44
MacBERTa + Att + CRF	86.79	84.12	85.36
M+DSCMG(Our)	**87.11**	**85.25**	**86.73**

On the CMEEE-V2, IMCS-V2-NER and CHIP-STS datasets, under identical parameter settings, the proposed model achieves the highest scores in precision (P), recall (R), and F1-score, thereby demonstrating its effectiveness and superiority in medical named entity recognition.

The experimental results indicate that M-DSCMG achieves strong performance on the three datasets, CMeEE-V2, IMCS-V2-NER, and CHIP-STS, with F1 scores of 65.26%, 80.31%, and 86.73%, respectively. These results are notably higher than those of baseline models, including BERT+CRF, BERT+BiLSTM+CRF, MacBERT+CRF, and MacBERT+Att + CRF.

These findings demonstrate the clear advantages of the proposed model in processing medical texts, particularly in terms of recognition accuracy and recall. This advantage primarily stems from the novel architectural design of the model, which integrates Dual-Stream Networks (DSN) and Cross-Stream Attention (CSA) alongside the fusion of Multi-Head Attention (MHA) and Gated Graph Neural Networks (GGNN).

The Cross-Stream Attention (CSA) mechanism facilitates interactions between character-level and word-level features, thereby enhancing entity boundary recognition. Meanwhile, the Multi-Head Attention (MHA) mechanism enables the model to more effectively capture entity importance and complex relationships, particularly in handling long-range dependencies and hierarchical semantic structures in medical texts. The GGNN architecture effectively models dependencies between medical entities, ensuring robust recognition performance even when processing long texts and complex syntactic structures.

The CRF decoding layer employed in this study further refines the sequence prediction of entity labels, enhances the global consistency of recognition results, and mitigates erroneous label transitions. The integration of GGNN and CRF enables the model to capture medical entity dependencies over a broader range and enhances its ability to accurately delineate entity boundaries.

### 4.3. Ablation experiment

To investigate the key factors that enhance the M-DSCMG method for medical named entity recognition and to verify whether the performance improvement primarily stems from the synergy of our proposed Dual-Stream Networks (DSN), Cross-Stream Attention (CSA), Multi-Head Attention (MHA), and Gated Graph Neural Network (GGNN), we conducted the following ablation experiments:

1) Removing the Cross-Stream Attention (CSA) and Dual-Stream Networks (DSN) while retaining Multi-Head Attention (MHA), GGNN, and CRF to compare the performance differences between models with and without CSA and DSN, thereby assessing their contributions.
2) Retaining DSN and CSA while removing Multi-Head Attention (MHA) to evaluate its role in the overall model and explore its impact on entity boundary recognition.
3) Retaining DSN, CSA, and MHA while removing GGNN to evaluate its contribution to modeling medical entity dependencies and analyze its importance in entity recognition.

**Fig 2:**
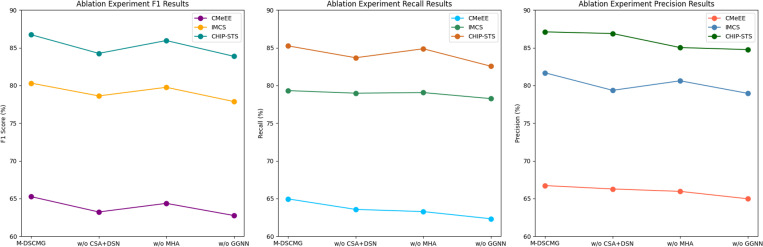
Ablation experiment results.

The ablation experiment results are presented in Fig 2. The experimental results indicate that the complete model (M-DSCMG) outperforms all ablation variants across all evaluation metrics, demonstrating that our proposed method effectively enhances medical entity recognition performance. After removing CSA, MHA, or GGNN, the model’s F1, Recall, and Precision scores decrease, indicating that the model’s overall performance relies on the synergy of multiple modules.

As shown in Fig 2, further analysis reveals that the removal of the cross-stream attention mechanism (CSA) and Dual-Stream Networks (DSN) leads to the most significant drop in the F1 score, suggesting that CSA plays a crucial role in integrating character-level and word-level information while refining entity boundary recognition. The removal of MHA (multi-head attention mechanism) results in a decrease in the F1 score, indicating that MHA enhances the model’s capacity to capture long-range dependencies, thereby improving its robustness in complex medical texts. Similarly, the removal of GGNN (gated graph neural network) leads to a decline in the F1 score, demonstrating that GGNN contributes to modeling the structured relationships among medical entities and facilitates the capture of contextual relevance. Each core module in the M-DSCMG method plays a vital role in enhancing model performance. Among them, CSA and Dual-Stream Networks (DSN) contribute the most, while GGNN and MHA also yield significant improvements. The experimental results robustly validate the effectiveness and superiority of our proposed model architecture in medical named entity recognition.

## 5. Discussion

Experimental results show that the proposed M-DSCMG (MacBERT + DSN + CSA + MHA + GGNN + CRF) method shows significant advantages on three datasets: CMEEE-V2, IMCS-V2-NER and CHIP-STS, especially in terms of precision, recall and F1 score, which comprehensively surpasses the existing mainstream methods. The performance improvement is mainly due to the innovative integration of the two-stream network structure (DSN) and the cross-stream attention mechanism (CSA), which realizes the effective fusion of character-level and word-level features, while reducing the negative impact of word segmentation errors on named entity recognition. The multi-head attention mechanism (MHA) further enhances the model’s ability to capture long-distance dependencies and improves the stability of processing complex medical terms; while the gated graph neural network (GGNN) plays a key role in modeling the dependencies between medical entities and optimizes entity boundary recognition. The ablation experiment results show that the synergy between CSA, DSN, MHA and GGNN is the key to improving the performance of the model, among which CSA and DSN contribute the most significantly, which emphasizes the importance of multimodal feature interaction in medical text processing. Compared with existing studies such as methods based on multi-granularity semantic dictionaries and methods that fuse global features with multiple local features, this study shows unique advantages in the flexibility and robustness of feature interactions.

Although the proposed model shows excellent performance, there are still some limitations and challenges. First, the model exhibits high computational complexity due to the inclusion of multi-head attention and gated graph neural network structures, resulting in relatively long inference time. In practical applications, it may be necessary to optimize computational efficiency. Second, although the DSN structure can partially alleviate the impact of word segmentation errors, the problem of blurred recognition boundaries still exists in some complex medical expressions, especially for rare medical terms. The current experiments are only conducted on Chinese medical text datasets, and the cross-lingual generalization ability of the model has not been fully verified. In resource-poor environments, the current method’s reliance on a large amount of annotated data and computing resources also poses an implementation barrier. To address these challenges, future research can explore the fusion of pre-trained medical knowledge graphs to enhance entity boundary recognition capabilities, extend the method to multilingual medical texts to evaluate its generalizability, and combine prompt learning and contrastive learning strategies to reduce reliance on large-scale annotated data.

In summary, the proposed M-DSCMG model shows excellent performance in the task of medical named entity recognition, provides an effective solution for the automatic parsing and information extraction of medical texts, and lays a solid foundation for downstream applications such as intelligent medical question answering and clinical decision support. The contributions of this study are:

1) A novel method combining a two-stream network structure with a cross-stream attention mechanism is proposed to effectively integrate character-level and word-level features;
2) The combination of multi-head attention and gated graph neural network enhances the model’s ability to handle complex medical terms and model inter-entity relationships;
3) The effectiveness of the method is verified through comprehensive experiments, and a detailed ablation analysis is provided to reveal the contribution of different components.

Despite the challenges of computational complexity and specific medical expression recognition, these limitations are expected to be improved in future work with the further development of techniques such as prompt learning, contrastive learning, and knowledge graph fusion, thereby further improving the efficiency and accuracy of medical text processing.

## 6. Conclusion

This paper introduces M-DSCMG, a model integrating MacBERT, Dual-Stream Networks (DSN), Cross-Stream Attention (CSA), Multi-Head Attention (MHA), Gated Graph Neural Networks (GGNN), and Conditional Random Fields (CRF), designed to address word segmentation errors, ambiguous entity boundaries, and challenges in capturing long-range dependencies in the Chinese Medical Named Entity Recognition (CMNER) task. Experiments on three medical text datasets—CMEEE-V2, IMCS-V2-NER, and CHIP-STS—demonstrate that the proposed M-DSCMG model surpasses existing state-of-the-art methods in precision, recall, and F1 score, confirming its effectiveness in medical named entity recognition. Ablation studies further indicate that the synergy among CSA, MHA, and GGNN plays a pivotal role in enhancing model performance. The proposed M-DSCMG model offers an effective solution for medical text information extraction, with potential applications in clinical decision support, intelligent medical question answering, and automated processing of electronic medical records. Moreover, it introduces novel perspectives and practical approaches for advancing medical information processing.
